# Identification of an Efficient Gene Expression Panel for Glioblastoma Classification

**DOI:** 10.1371/journal.pone.0164649

**Published:** 2016-11-17

**Authors:** Thomas J. Crisman, Ivette Zelaya, Dan R. Laks, Yining Zhao, Riki Kawaguchi, Fuying Gao, Harley I. Kornblum, Giovanni Coppola

**Affiliations:** 1 Semel Institute for Neuroscience & Human Behavior and Department of Psychiatry and Biobehavioral Sciences, University of California Los Angeles, Los Angeles, California 90095, United States of America; 2 Department of Biological Chemistry, University of California Los Angeles, Los Angeles, California 90095, United States of America; 3 Eli and Edythe Broad Center of Regenerative Medicine and Stem Cell Research, University of California Los Angeles, Los Angeles, California 90095, United States of America; 4 Jonsson Comprehensive Cancer Center, University of California Los Angeles, Los Angeles, California 90095, United States of America; Swedish Neuroscience Institute, UNITED STATES

## Abstract

We present here a novel genetic algorithm-based random forest (GARF) modeling technique that enables a reduction in the complexity of large gene disease signatures to highly accurate, greatly simplified gene panels. When applied to 803 glioblastoma multiforme samples, this method allowed the 840-gene Verhaak et al. gene panel (the standard in the field) to be reduced to a 48-gene classifier, while retaining 90.91% classification accuracy, and outperforming the best available alternative methods. Additionally, using this approach we produced a 32-gene panel which allows for better consistency between RNA-seq and microarray-based classifications, improving cross-platform classification retention from 69.67% to 86.07%. A webpage producing these classifications is available at http://simplegbm.semel.ucla.edu.

## Introduction

Glioblastoma (GBM) is the most common and most fatal form of primary malignant brain tumor. The survival rate with treatment is frequently under two years, with the median survival rate being 12.2 months without treatment [1]. GBMs are highly heterogeneous and show highly variable gene expression patterns. Several classification schemes have tried to capture this variability by using gene expression data in an attempt to identify more homogeneous sub-categories for prognosis and drug testing [1,2].

The most commonly used classification scheme was proposed by Verhaak et al. in 2010, and divided GBMs into Proneural, Classical, Neural, and Mesenchymal types based on gene expression measured with microarrays. These subcategories differed both in terms of median survival rates, which were highest (13.1 months) in the Neural and lowest (11.3 months) in the Proneural type [1], and in response to aggressive treatment (defined as requiring more than 3 courses of chemotherapy). In the original study aggressive treatment was significantly more beneficial in the Classical and Mesenchymal subtypes, and least effective in the Proneural subtype [1].

The Verhaak et al. classification algorithm was developed by applying a centroid-based classifier, 'ClaNC' [3], on a microarray dataset of 200 GBM samples. Using 173 of the 200 samples (described as ‘core’ samples by Verhaak et al.) and a linear discriminant analysis (LDA) method of gene selection and variable reduction, ClaNC was used to build a 4 subcategory classifier and assign a category to each of the 200 samples [1].

The Verhaak et al. classifier utilizes 210 genes per GBM category, resulting in the classifier being based on 840 total genes. Since testing hundreds of genes in order to classify GBM samples is impractical outside of large-scale microarray and RNA-sequencing experiments, we set out to identify a reduced gene set that would allow classifications to be made with a subset of genes while retaining classification accuracy.

To accomplish our goal of producing a method of selecting a significantly smaller subset of genes which recapitulates the Verhaak et al. GBM subclassifications, we have developed a method of variable reduction in random forest models designed to reduce the complexity of the classifier while maintaining accuracy. Our approach uses a novel method of random forest (RF) variable reduction based loosely on a genetic algorithm (GA) designed by Waller et al. [4]. This iterative GA framework rewards genes based on expression or other variables from the best randomly-selected subsets by allowing them to continue to the next generation of subsets. Using this approach, variables which do not perform as well in random pairings are eliminated. The final result of our utilization of this algorithm is a set of 48 genes (GBM48 panel) which is highly accurate in assigning Verhaak et al. categories in a test set of 803 GBM expression samples collected from publicly available datasets. Additionally, we have used the same algorithm to maximize accuracy on RNA-seq based data creating a second GBM RNA-seq 32 gene panel. This 32 gene RNA-seq based panel greatly improves our ability to compare RNA-seq based classification to microarray based classification using the original 840 gene Verhaak et al. classifier. These findings provide a simpler subset of genes whose expression can be used for classification, as well as a general method whereby similar strategies may be employed in other systems to aid in reducing the complexity necessary to describe them.

## Methods

### The Verhaak et al. Classifier

The Cancer Genome Atlas (TCGA) training set used to build this model consists of 173 ‘core’ samples [1]. Each of these samples had genome-wide expression patterns collected from runs on three platforms (Affymetrix HuEx array, Affymetrix U133A array and Agilent 244K array). In order to improve reproducibility and to select genes behaving consistently across multiple array platforms, the total number of probes used in the analysis was reduced to 1,740 through a series of filters, described by Verhaak, et al. [1]. Briefly, the first filter was consistency across at least two of the three array platforms, which was calculated by comparing the expression pattern across all three platforms. If a gene had a 0.7 or higher correlation across two platforms, it was kept in the analysis. This filter resulted in 9,255 genes remaining from the original 11,861 unified gene expression patterns. The second filter was high variability across samples, i.e. probes with mean absolute deviation (MAD) greater than 0.5 across all patients were filtered out, which resulted in 1,903 genes remaining. The third filter removed genes where the individual MAD was significantly different than the averaged MAD, in order to remove genes with extremely variable standard deviation. These filters resulted in a final a set of 1,740 probes combined across three microarray platforms. These 1,740 genes were then used to estimate the correct number of clusters/classifications. This was done by attempting multiple different cutoffs and selecting the number of clusters with the highest stability using consensus average linkage hierarchical clustering [5]. These experiments resulted in 4 subclasses being determined as the optimal cutoff.

Using ClaNC, the total gene set to describe these 4 clusters/classifications was then reduced to 840 probes through linear discriminant analysis and cross-validation. The final set of 840 probes was then used to create the Verhaak et al. centroid-based classifier. These 840 genes constituted the starting pool of variables used for the algorithm presented here, aimed at the identification of an optimized smaller subset.

### Datasets Used in this Study

The six datasets used for the process of building and validating our algorithm are listed in Table 1 along with their relative sizes, platforms and final model accuracy. Additionally, we used 122 RNA-seq samples from TCGA generated using the Illumina HiSeq2000 v2 platform. These were used to produce a second reduced 32-gene RNA-seq panel for better RNA-seq/microarray data agreement on Verhaak et al. classifications.

**Table 1:** Datasets used in this experiment.

Dataset	N. of Samples	Usage	Platform	Accuracy
**1. TCGA Training Data**	171	Training Set	Affymetrix HT Human Genome U133 Array Plate Set	91.81%
**2. TCGA Test Data**	296	Test Set Used to Direct Training	Affymetrix HT Human Genome U133 Array Plate Set	91.55%
**3. GDS4470**	46	Test Set Used to Direct Training	Affymetrix Human Genome U133 Plus 2.0 Array	89.13%
**4. GDS4477**	27	Test Set Used to Direct Training	Affymetrix Human Genome U133 Plus 2.0 Array	81.48%
**5. GDS4467**	35	Test Set Used to Direct Training	Affymetrix Human Genome U133 Plus 2.0 Array	91.43%
**6. Rembrandt (GSE68848)**	228	Hold Out Test Set	Affymetrix Human Genome U133 Plus 2.0 Array	93.86%
**Total**	803			90.91%

Table 1 shows the datasets used for the development of our optimized model as well as the final classification accuracy of our final GBM48 panel classifier. Random models were built using the 171 TCGA 'core' training samples, and evaluated on the 575 samples from the TCGA, GDS4477, GDS4470 and GDS4467. The best model/models from each run were then tested against the Rembrandt dataset (GSE68848) to test for over-training.

All 6 datasets used were obtained using Affymetrix microarrays, though different versions of microarray chips were used depending on the dataset. TCGA data was downloaded from the TCGA website [6], the Rembrandt data from the Rembrandt data download page [7] and the other datasets were downloaded from the Gene Expression Omnibus repository [8,9], including datasets from Sturm et al. [10] consisting of 46 samples, Schwartzentruber et al. [11] consisting of 27 samples and Grzmil et al. [12] consisting of 35 samples (Table 1). When more than one probe existed for a given gene in a given dataset, prior to normalization and batch effect adjustment the brightest probe was selected to represent each individual gene.

All datasets were combined and normalized using the R package limma [13], and batch effects were adjusted using ComBat [14]. ClaNC was used to create a centroid-based classifier [1], and a Verhaak et al. category (Mesenchymal, Proneural, Neural, or Classical) was assigned to each sample within the test sets.

A subset of the TCGA data consisting of 122 samples, for which both RNA-seq and microarray data were available, was used to develop our RNA-seq panel. This subset was used to find a small group of genes with high information content and correlation with microarray data in order to improve RNA-seq to microarray classifications.

### Optimal Gene Cutoff Selection

**Fig 1:**
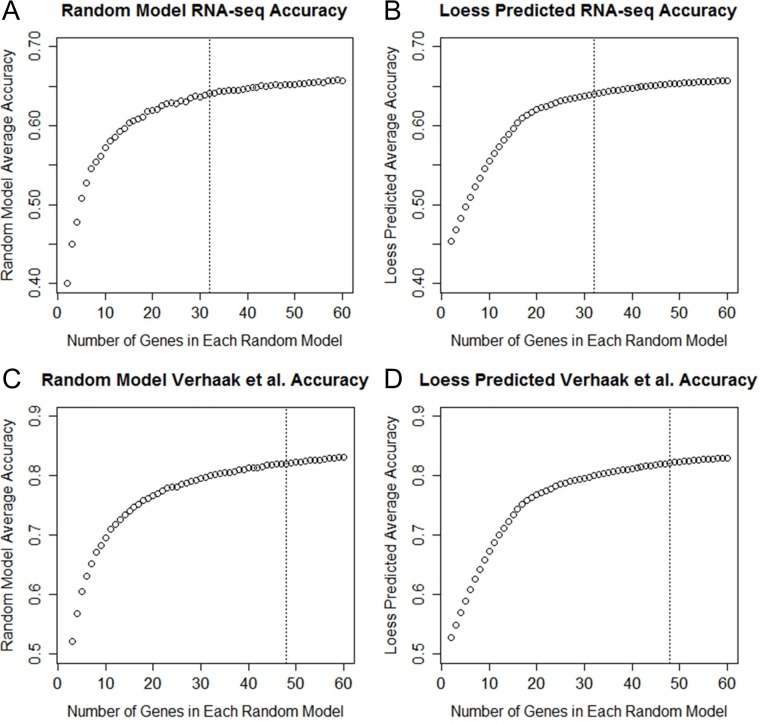
Random Model-Based Panel Size Determination. Figures show different average values of models made with random gene subsets of between 2 and 60. Each data point in A and C represent the average comparative accuracy of the 1000 random models on all 803 samples. The models themselves were built using the randomly selected genes trained with only the samples used in the Verhaak et al. study. In B and D, these figures are smoothed curves produced from fitting of the random data using local regression. By smoothing the curves, a more accurate guess can be made as to how much data is likely being added with larger gene subsets. For the purposes of this study we determined an appropriate cutoff to be when an additional gene adds less than 0.001 percent to average random model accuracy. The final values selected using this approach were 32 genes for RNA-seq data (A and B) and 48 (C and D) for the reduced Verhaak et al. classification. Both these cutoffs are marked with a dotted line connecting to the x axis.

The first step in our analysis was to estimate the optimal size with which to produce a classifier. In order to do this, we produced 1000 random forest models from randomly selected genes at cutoffs of between 2 and 60 gene subsets for both the reduced GBM48 classification panel and for the reduced RNA-seq panel. We then estimated the average random model accuracy on all 803 samples. The resulting curve was fitted using local polynomial regression fitting, and additional probes were included only if they improved the average model by at least 0.001% (Fig 1) [15]. This produced an optimal cutoff of 48 genes for our reduced Verhaak et al. panel and 32 genes for our improved RNA-seq panel.

### Feature Selection

Our initial expression set comprised the Affymetrix expression levels for the 840 Verhaak et al. probes in 171 of the 173 ‘core’ samples (2 samples from the original training set were not available for download from the TCGA website). We then included only genes existing with the same gene symbol across all six training and test sets, which resulted in a total starting pool of 753 probes.

We used five datasets to build the classifier (datasets #1–5, Table 1) and then used the Rembrandt [7] test set (dataset #6) for final verification. Each subset of 48 genes was trained on the TCGA training dataset (dataset #1) and evaluated for fitness on datasets #2–5 but not on the Rembrandt dataset (dataset #6), in order to avoid overfitting. This process helped to select gene subsets that worked on more than one Affymetrix chip type and on all datasets used in the subset fitness evaluation process. The final model was validated on the independent Rembrandt dataset (#6) that was not used to build the classifier in the training process. The Rembrandt dataset was selected for this purpose because it was the largest test dataset from a source other than the original classifier.

### Random Forest

The "Random Forest" algorithm was named as such because it consists of hundreds or thousands of decision trees. The consensus of the classifications predicted by these decision trees represent the classification predicted by the overall model. For our random forest models, we used the "randomForest" library [16], an R implementation based on the original design by Leo Breiman [17]. All random forest models were built identically with genes selected from varSelRF, RFE and GARF. We used 5000 trees per model with a default number of variables randomly sampled at each node/split of the decision trees.

### Genetic Algorithm/Random Forest (GARF) Approach

**Fig 2:**
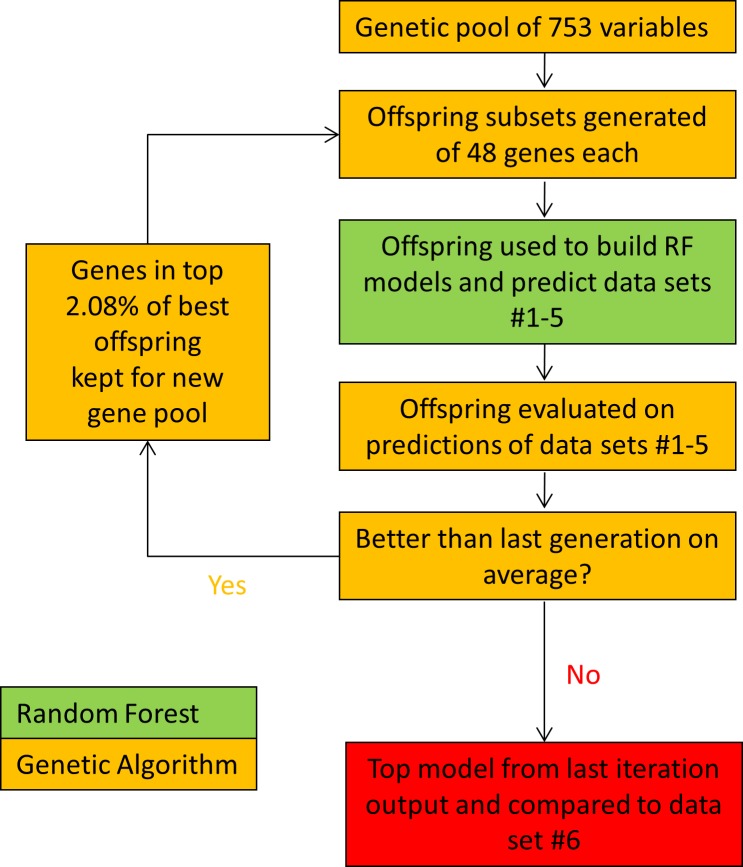
Genetic Algorithm/Random Forest Flow Chart. General description of our Genetic Algorithm/Random Forest approach used to select the best 48-gene classifier. A starting gene pool is refined by removing the least "fit" genes until a subset remains representing a local maximum based on the starting subsets of genes selected.

Genetic algorithms (GA) are algorithms that behave similarly to natural selection. The basis of the genetic algorithm used here is inspired by a small molecule variable optimization algorithm based on linear regression found in Waller et al. [4]. Our genetic algorithm starts with a pool of fixed or variable randomly-selected variables from which ‘offspring’ models can be created. An iterative algorithm, our GARF approach slowly eliminates less fit variables by keeping only the variables that make up the best models. This leads to fitter generations of offspring models with each subsequent generation (Fig 2).

Our algorithm starts off by selecting offspring subsets that are selected by the subset selector "sample" from the base R package [18]; random forest models are then built on these subsets and are evaluated on datasets #1–5 for fitness (described in the ‘GARF Offspring Fitness Evaluation’ section). The genes in the best subsets are allowed to move on to the subsequent generation. The variables themselves are treated as a pool, where each subsequent generation of 48 gene subsets is randomly selected from the current pool.

The first key difference when compared to the Waller et al. approach is that instead of removing the least fit variables, we keep the variables in subsets that produce the best models. This difference in methodologies only removes variables which do not appear in the best models, rather than punishing variables that are potentially grouped with other poorly performing variables. This eliminates the need for the ‘taboo’ search found in the original algorithm, which confirms at every elimination step that genes in the least fit models are not present in any highly fit models.

Furthermore, in the case of our algorithm, the cutoff is dynamically selected based on the number of variables in the pool. This cutoff is set to keep the number of models needed to allow all genes to remain in the pool; thus, theoretically, if all genes perform the same, they should all be kept in the next iteration. This ensures that only genes which appear in more than a single top model will move on to the next generation. The Waller et al. algorithm allows a static kill factor to be used, with a suggested value of 5% of the worst models excluding variables found in top models. In contrast, our approach favors keeping variables which have combined predictive value together in the pool, allowing greater opportunities in subsequent generations for them to be placed together again with other groups with higher predictive value. This permits optimization to occur as the result of genes which together describe the system well, rather than removing individual genes which did not describe the system well or which may simply not have a strong effect on the total value of the subset of variables in a given model.

The third difference is that Waller et al. utilize leave–one-out cross-validation to establish fitness. Grouping together multiple cohorts and building the model on samples from multiple cohorts, and then utilizing cross-validation to validate the model increases the possibility of over-training. Given that reproducibility is a challenge both between platforms and labs, our algorithm is designed to train on a single cohort, but then only select genes with reproducible effects in alternative cohorts. Building a model on a single cohort and being able to replicate it independently on additional cohorts improves the likelihood that it will be reproducible on additional datasets using a similar platform.

### GARF Offspring Fitness Evaluation

To test the fitness of each subset, a random forest model is built using the genes that make up that subset. The fitness of each subset is then evaluated by how many identical classifications the random forest model built on it produces when compared to the Verhaak et al. ClaNC classifications obtained using all genes. The overall fitness is the average accuracy produced by all subset-derived random forest models built in a generation of subsets. The fitness evaluation process excludes dataset #6 which is used for final model validation after the completion of the algorithm in order to evaluate if the model has predictive capabilities outside of the datasets it was trained on.

### GARF Runs

The first generation of our genetic algorithm starts by constructing the number of subsets equal to the total number of genes in the starting gene pool. In the case of this system, we start with 753 genes, and 753 subsets of 48 genes are created in the first generation of models. The number of created subsets and random forest models built on those subsets in each subsequent generation is equal to the number of genes remaining in the pool. This ensures the number of models constructed reduces as the number of possible subsets shrinks due to the shrinking size of the pool of variables.

At each iteration, average model accuracy is evaluated by comparing the Verhaak et al. model built with ClaNC and the subset-based random forest model predictions against the 575 samples in datasets #2–5 (Table 1). If the classification of the random forest model is identical to the classification predicted by the Verhaak et al. model, the prediction is considered to be correct.

At each successful iteration at which average model accuracy improves, the unique genes from the top 2.08% of models from that iteration move on to form the starting pool for the next generation. The 2.08% cutoff is determined as the minimum number of models needed in order to ensure that 100% of the genes have the possibility to survive to the next generation of offspring models. The percentage kept can be described with the following formula: (100% / # of genes in model). For example, if there were 400 genes in the pool, 400 subsets for building models would be made, 9 (400/48 = 8.32) of which would move on to the next generation. These 9 models would contain 432 genes if there was no overlap, making this the minimum number kept to allow all 400 genes to potentially move on to the next generation.

This iterative process ends when the next generation of models fails to be superior to the previous generation in average model accuracy or when the target number of genes is reached in the pool of variables. If the next gene pool fails to improve upon the last, the genetic algorithm stops and the best-scoring models from the best-performing variable pool are selected for follow-up with the holdout test set (dataset #6).

The entire process, starting from the initial pool of 753 genes, was run 10 times, and what is reported in the results section was the best model from all 10 runs as ranked by comparison across all samples included in the test set.

### VarSelRF

Variable selection using random forests (varSelRF) is a variable reduction package which utilizes random forest models [19]. We use it here as the first of two comparisons to our own novel variable reduction method. One thousand runs of varSelRF were run using 5000 for the number of initial trees, 2000 trees in each iteration, and 0.2 was used for the number of variables to drop at each iteration. The average number of genes per run was 105.88; the maximum number of genes in a varSelRF output was 527, and the minimum was 15 with a standard deviation of 87.06 variables. Accuracy across the different datasets is reported in S1 Table.

### RFE

Recursive feature selection (RFE) is a variable reduction package which is part of the R ‘caret’ package [20]. We use it here as the second of two comparisons to our own novel variable reduction method. As a comparison, one thousand runs of RFE were carried out at a cutoff of 48 variables. The output, though comparable to varSelRF, had a much lower standard deviation, and was significantly more consistent across all 1000 runs. Accuracy across the different datasets is reported in S2 Table.

## Results

The number of possible groups of 48 genes from the 753 genes that made up our starting pool is greater than 2.13 x 10^76 [21]. As a result, testing every possible combination would take more than 5 x 10^68 years using a single thread on a core i5 processor. Therefore, a variable reduction technique was needed to reduce search space while improving the accuracy of the gene selection for these models.

We first used varSelRF, a commonly used algorithm for gene selection in random forest models [19]. Unlike the method reported here, varSelRF cannot be set to run at certain gene subset cutoffs, and the ability to select an optimal number of variables has been reported as being a benefit of varSelRF [22]. We ran varSelRF 1000 times and tested the genes it selected in RF models on all 803 samples, then tested the accuracy on dataset #6 consisting of 228 samples and on datasets #1–5 consisting of 575 samples used as training sets. The closest comparison to our method were models which showed up using an optimum cutoff of 43 and 62, which represented 111 of 1000 of our varSelRF runs. The average of such models was 83.27% and the best varSelRF model in that range was 85.68% (62 genes), the worst being 81.82% (43 genes). By comparison, our best random model at 48 genes had 87.65% accuracy, about 2% better than the best varSelRF runs in the same range. Average models with 43 randomly selected genes were competitive with varSelRF at 82.13% (compared to 81.82% for models selected by varSelRF with 43 genes). The best model from the 1000 varSelRF runs was 88.54% accurate, and required 258 genes.

Our second comparative analysis was carried out using RFE from the caret package. We ran RFE 1000 times using 10-fold cross-validation. Using the top 48 genes from each run, we then built a random forest model on our training set using the selected genes, and evaluated them against all test sets. On average, the RFE output across all test and training sets (803 samples) resulted in an accuracy of 84.38%. The best suggested output from RFE resulted in an accuracy of 87.55%, making it outperform varSelRF in the general range of our optimized model. The worst model was 81.57% (S2 Table).

We also produced 1000 random forest models built on 48 randomly selected genes. These ‘random’ models produced an overall accuracy of 82.04%, and the best random model had 86.55% accuracy. RFE outperformed the random models by more than two percent on average, and one percent for the best model output by RFE (87.55% vs 86.55%). The best randomly generated models outperformed the best varSelRF model in our range of interest: 85.68% with 62 genes for VarSelRF vs 86.55% for the best random model. On average, varSelRF was slightly better on average than the random models in our range of interest, 83.27% on average for suggested gene subsets between 43 and 62 genes (compared to 82.04% at random). Our GARF approach significantly outperformed all three.

After our cutoff of 48 genes was selected to be the optimum number for our purposes (see **Optimal Gene Cutoff Selection**), we then created random offspring subsets of 48 genes from the variable pool. These subsets were used to create random forest models to predict the Verhaak et al. classifications. These classifications were then compared with the original classification. Models that performed poorly had their genes eliminated from the overall pool of variables used to make the next generation. With every generation, variables were iteratively removed from our pool of variables based on fitness, eventually resulting in a vastly improved subset over random variable selection (Fig 2). The final GBM48 panel achieved 90.91% accuracy for all 803 samples when considering the original Verhaak et al. ClaNC classification [1]. For the classifications based on ClaNC, Mesenchymal was the largest subtype with 243 (30.26%), Classical was the second largest with 210 (26.15%), 204 (25.40%) of the samples were Proneural, and Neural was the smallest classification with 146 (18.18%) samples. For classifications based on the random forest model using our GBM48 panel, Mesenchymal was the largest subtype in the 803 samples with 268 (33.37%) samples, followed by Proneural, Classical and Neural with 206 (25.65%), 200 (24.91%), and 129 (16.06%) samples, respectively.

Our own models based on our GARF algorithm significantly outperformed VarSelRF on average by over seven percent in our range of interest, and on average by over six percent when VarSelRF was able to select its own optimal cutoff. Our GARF approach out-performed the best model produced by RFE by more than three percent.

### Survival Analysis

**Fig 3:**
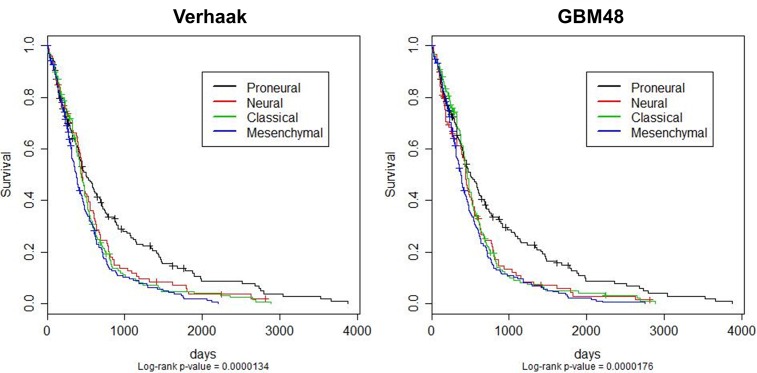
Kaplan-Meier Survival Curves for our Combined Cohort. Kaplan-Meier survival curves for 537 patients from the Rembrandt and TCGA datasets, classified using the original Verhaak et al. ClaNC-based classification (left) and our reduced random forest classification based GBM48 panel described in this paper (right). The y-axis represents the proportion of surviving patients. Both classifications show a statistically significant difference between Proneural and the other subtypes according to the log-rank (p-values at the bottom of the figures). Our GBM48 panel shows more significant differences in expected clinical outcome.

In order to explore survival by subtype in GBM and to examine the similarity in survival of the two alternative models (the panel based on the GBM48 method and the original classifier), Kaplan-Meier survival curves were generated for all 537 samples with available survival data from the 803 samples used in this study (Fig 3). Our new reduced classification scheme based on the GBM48 panel and the classifications based on the original 840 gene classifier both support the idea that the Proneural subtype is associated with a better outcome. Both our classifier and the original classifier had similar p-values associated with the differences in the significance of survival outcome between subgroups. Our classifier showed a similar degree of variance from outcomes when compared to the original classifier with a p-value of 1.76 x 10^-5 in comparison to a p-value of 1.34 x 10^-5 for the original classification technique. This suggests that our simpler classifier has a similar clinical significance as the original classification technique with regard to survival.

### Genes Selected by Our Model

Our best model consisted of the genes listed in Table 2, which also shows average normalized and batch effect-adjusted expression of each gene in each classification along with their standard deviations (SD). Their final accuracy across all 803 samples was 90.91%. The individual set-by-set accuracy breakdown is listed in Table 1. Overall the GBM48 panel was 92.65%, 81.51%, 89.05% and 96.71% accurate in predicting the Proneural, Neural, Classical and Mesenchymal subtypes, respectively.

**Table 2:** GBM48 Average Expression by Classification.

Gene	Proneural	SD	Neural	SD	Classical	SD	Mesenchymal	SD
**ACSL3**	4.14	1.06	4.97	1.25	5.99	1.03	4.62	1.16
**ACYP2**	4.78	1.17	6.12	0.83	5.12	0.91	4.34	1.02
**ADCY9**	3.54	0.71	2.99	0.76	3.64	0.64	3.52	0.65
**ARPC1B**	3.88	1.25	4.55	0.94	4.33	0.87	6.07	1.06
**BCAN**	4.84	1.31	4.07	1.08	4.41	1.16	2.32	1.30
**BCL7A**	4.48	1.05	3.33	0.78	3.37	0.78	2.58	0.74
**CASP5**	2.60	0.70	2.87	0.84	2.86	0.66	3.23	0.83
**CDC25A**	3.62	0.88	2.87	0.72	3.15	0.66	2.85	0.76
**CHD7**	5.62	1.38	3.39	1.07	4.11	0.96	3.06	1.17
**CLASP2**	5.57	0.98	5.23	0.87	4.38	0.93	3.63	1.04
**COL5A1**	3.18	1.40	2.86	1.12	3.63	1.18	5.03	1.46
**CREB5**	4.02	1.42	3.05	1.22	4.58	1.12	3.10	1.07
**CSPG5**	5.18	1.23	5.21	1.04	5.59	1.11	3.25	1.45
**DCX**	6.76	1.65	3.95	1.94	3.19	2.19	2.42	2.11
**DDX42**	4.55	0.85	3.70	0.87	4.45	0.75	4.12	0.83
**EPHB4**	2.63	0.77	3.01	0.68	3.66	0.58	3.49	0.71
**FEZF2**	3.03	1.32	3.57	1.54	2.67	0.88	2.70	0.99
**FGFR3**	2.76	1.07	4.31	1.28	4.74	1.23	3.21	1.31
**FNDC3B**	4.15	1.05	3.61	1.02	4.93	0.89	5.50	0.91
**FZR1**	2.96	0.54	2.76	0.48	3.28	0.45	2.86	0.48
**GSK3B**	5.01	0.85	3.82	0.71	4.35	0.77	4.11	0.88
**GTF2F1**	3.94	0.77	3.52	0.79	4.37	0.60	3.72	0.75
**IL1R1**	2.79	1.08	2.83	1.11	2.89	0.99	4.45	1.06
**LAPTM5**	4.83	1.58	5.49	1.23	4.81	1.17	6.32	1.15
**NDRG2**	4.07	1.45	5.18	0.99	4.44	1.22	2.98	1.32
**NR2F6**	2.92	0.58	2.89	0.52	3.33	0.44	3.11	0.53
**PAK3**	5.17	1.45	4.17	1.65	2.82	1.40	2.71	1.42
**PIPOX**	2.68	1.25	4.66	1.22	5.43	1.00	4.18	1.18
**PMP22**	4.84	1.41	6.00	1.18	6.57	1.13	6.13	1.00
**PPM1D**	4.68	0.92	4.25	0.89	4.19	0.83	3.74	1.05
**PRPSAP2**	5.09	1.00	5.22	0.81	4.47	0.75	3.96	0.93
**PTPRC**	3.23	1.37	4.36	1.08	3.37	1.05	4.98	1.06
**PURG**	2.26	1.08	2.52	0.99	2.61	0.90	2.32	0.97
**RGS12**	3.01	0.66	2.98	0.60	3.48	0.58	2.73	0.57
**SH3GL3**	3.50	1.10	3.65	1.22	2.27	0.87	2.68	1.06
**SHC1**	3.47	0.91	3.63	0.75	4.14	0.83	5.06	0.98
**SSH3**	2.66	0.78	3.45	0.57	3.53	0.58	3.42	0.64
**TIMP1**	4.85	1.99	5.80	1.60	6.65	1.30	7.45	1.20
**TMBIM1**	2.95	1.15	4.67	0.79	4.18	1.00	4.73	0.75
**TMEM43**	4.31	0.88	4.16	0.85	4.65	0.89	4.90	0.81
**TRIB2**	4.79	1.69	4.45	1.52	6.06	1.10	4.11	1.30
**TRRAP**	3.91	0.88	3.07	0.99	4.21	0.70	3.67	0.88
**UROS**	4.15	1.03	4.77	1.05	3.58	0.81	3.98	0.98
**VAX2**	4.04	0.92	2.98	1.27	2.99	0.81	2.55	0.77
**WASF1**	5.86	1.23	4.90	1.33	3.98	1.10	3.71	1.08
**ZDHHC18**	3.15	0.63	2.99	0.70	3.44	0.58	3.46	0.60
**ZEB2**	4.65	0.99	3.81	0.95	3.56	0.78	3.66	0.87
**ZNF446**	3.22	0.67	3.38	0.65	3.73	0.64	3.17	0.63

Table 2 shows gene symbols from the 48 genes from our top model. Average normalized and batch effect-adjusted expression levels for each gene are shown for each classification along with the standard deviation for each directly in the column to the right.

**Fig 4:**
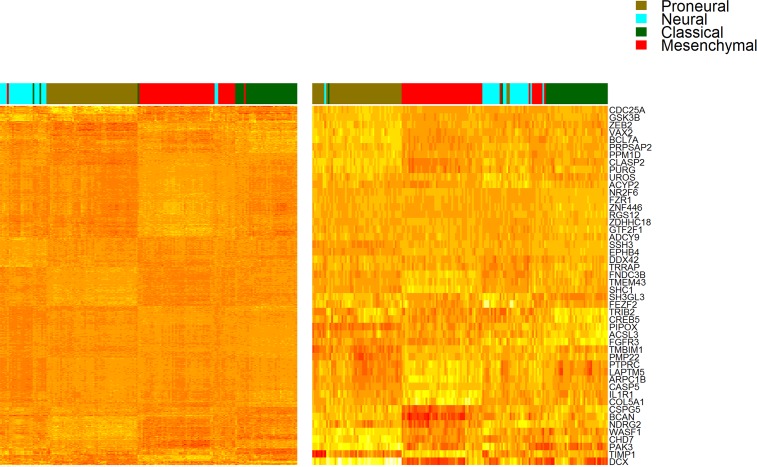
Heatmap Comparing GBM48 and Verhaak et al. Classifier. Heatmap and hierarchical bi-clustering of all 840 Verhaak et al. genes (left) and the GBM48 panel genes (right). GBM48 gene names are listed on the right.

In order to compare the similarity of our GBM48 panel to the 840 Verhaak et al. genes, we created a heatmap and clustering of our simplified panel and compared it to a corresponding heatmap including all Verhaak et al. genes (Fig 4). The sample separation into the four Verhaak et al. subtypes was very similar, supporting the use of GBM48 to achieve Verhaak et al. classification.

**Fig 5:**
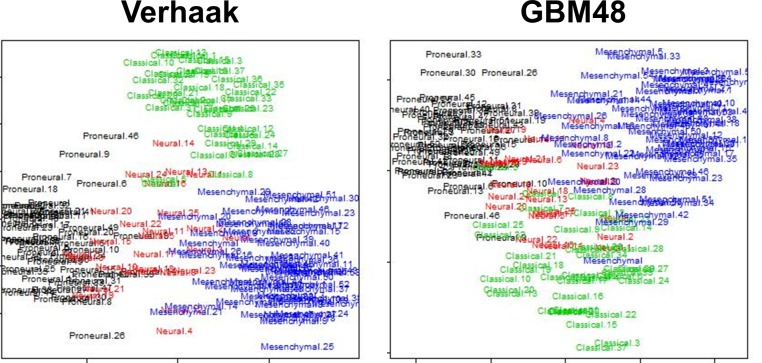
Multidimensional Scaling Plot Comparing GBM48 and Verhaak et al. Classifier. Multidimensional scaling of 173 core TCGA samples based on 840 Verhaak et al. genes (left) and the GBM48 panel genes (right).

Similarly, multidimensional scaling (MDS) was used to cluster samples based on either the 840 Verhaak et al. genes used for the original classifications or the GBM48 panel (Fig 5). Though there appears to be a clear separation into Proneural, Mesenchymal, and Classical subclasses, it is worth noting that the Neural subclass tended to be similar to all three other subclasses in the Verhaak et al. classifications. In addition, healthy control samples in the Verhaak et al. study were classified as Neural, suggesting that this subtype is representative of the lack of extreme variation characterizing the other subtypes.

We annotated the GBM48 panel using string-db.org [23], GOrilla [24] (S1 Fig), g:profiler [25,26] and Gene Set Enrichment Annotation (GSEA) [27,28] packages.

The GSEA analysis resulted in a statistically significant association with Alzheimer’s disease associated genes. A total of 14 of the GBM48 panel’s genes associated with upregulation (FDR-adjusted p-value of 3.92 x 10^-6) and 11 of the GBM48 panel’s genes associated with downregulation (FDR-adjusted p-value of 7.76 x 10^-5) in Alzheimer’s disease. Additionally, two promoter motifs corresponding to *SP1* and *POU2F1* were found to be enriched in the GBM48 set. The first promoter region was linked with 15 of the GBM48 panel’s genes which contained the GGGCGGR motif. This motif has a statistically significant relationship with *SP1* (FDR-adjusted p-value of 2.55 x 10^-4), a zinc finger transcription factor associated with cellular processes including cell growth, differentiation and apoptosis [29], which has been shown to be upregulated in gliomas with poor clinical outcome[30]. The second promoter region was linked with 6 of the GBM48 panel’s genes which contained the CWNAWTKWSATRYN motif for the *POU2F1* transcription factor (also known as Oct-1, FDR-adjusted p-value of 4.73 x 10^-4), which has been shown to be differentially expressed in human glioblastoma cells [31,32].

G:profiler reported a number of statistically significant gene ontology associations with this dataset largely related to anatomical structural differentiation of various nervous tissues, chemotaxis, axonal guidance and protein kinase binding (S1 Fig). For protein kinase binding across the entire GBM48 panel, 9 of the genes were associated with an FDR-adjusted p-value of 1.82 x 10^-2. Additionally, two regulatory motifs were identified; one for transcription factor AP-2α (GSCCSCRGGCNRNRNN) was found in 20 of the GBM48 panel’s genes. The transcription factor AP-2α has been shown to be downregulated in gliomas and is believed to be negatively associated with the grade of human glioma [33,34]. The second was identified for EGR-1 (GCGGGGGCGG), which was found in 26 of the GBM48 panel’s genes. The transcription factor EGR-1 is a zinc finger protein that has been shown to be suppressed in human gliomas and human glioblastoma cell lines [35,36]

**Fig 6:**
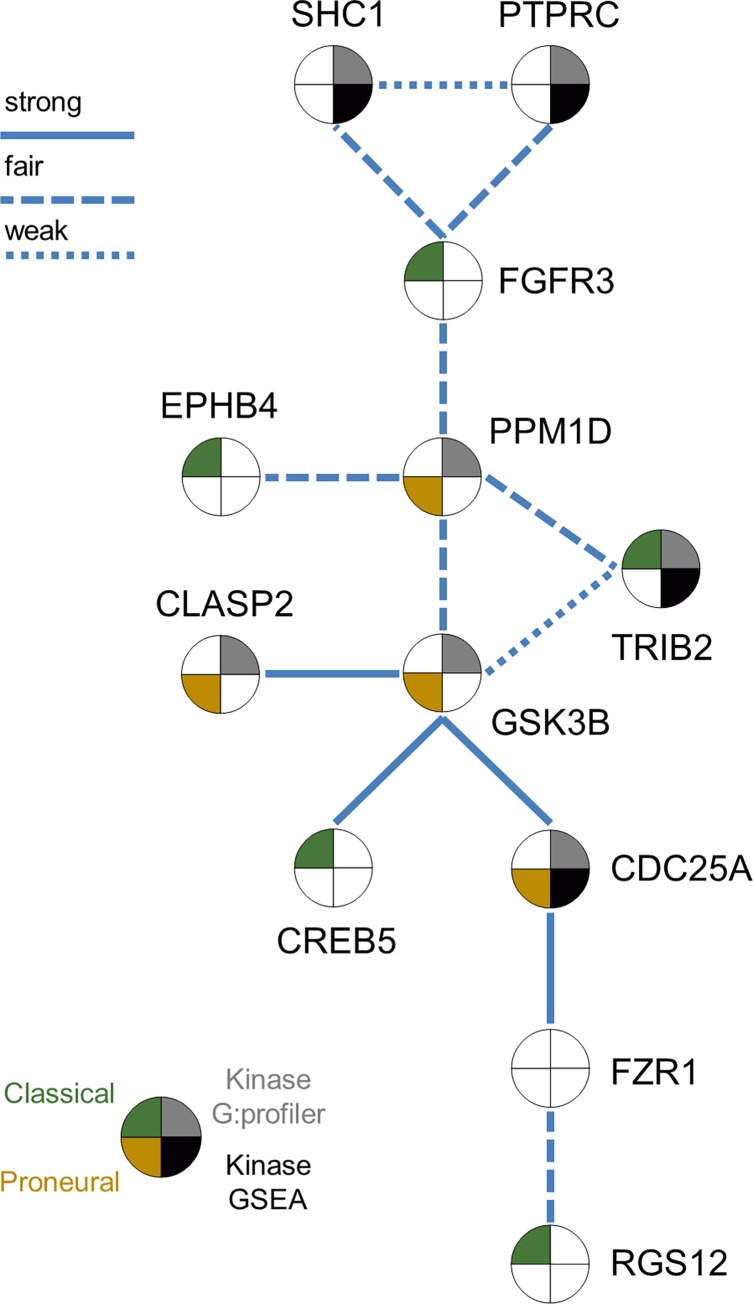
Experimentally Validated Gene Network in the GBM48 Panel. Experimentally associated gene network from string-db with statistically significant gene set enrichment sets from G:profiler and GSEA. Two of the four Verhaak et al. subclassifications (Classical and Proneural) were over-represented in our experimentally associated gene set from our GBM48 panel. Five of the 12 genes in the network were biomarkers for the Classical Verhaak et al. subset and represented in green, four genes were biomarkers for Proneural and are represented in this figure in gold. Genes which were linked with the statistically significant over-enriched kinase binding and activity pathways are represented in this figure in grey for the genes described in the G:profiler database and black for the genes described in the GSEA database, representing seven and five genes from the total gene network respectively.

Twelve of the GBM48 panel’s genes were found to be associated with each other by way of experimental evidence according to String-db (Fig 6). Amongst these 12 genes, 7 were associated with protein kinase binding in G:profiler (p-value 1.77 x 10^-5) and 4 of the 12 were found to be associated with kinase binding and transferase activity in GSEA (p-value 2.34 x 10^-4). Also according to GSEA, 5 of the 12 genes were associated with the Classical tumor subtype, and 4 of the 12 were associated with the Proneural subtype. The genes involved in this network are shown in Fig 6 and are color-coded to represent which of the subtypes (Classical or Proneural) they were associated with, as well as which database described them as being statistically significantly related to protein kinase binding (either G:profiler or GSEA).

### RNA-seq Comparison and Panel Optimization

In order to test the consistency between microarray- and RNA-seq-based expression data, we tested our method on 122 samples for which both were available. Importantly, the traditional Verhaak et al. classification scheme using 793 genes which were present in both RNA-seq and microarray data produced the same classification in 69.67% (85/122) of the samples.

Following this analysis, our GARF method was run using the core TCGA samples as the training set and the RNA-seq data as the test set. After normalization and correction of batch effects, we attempted to find the ideal gene signatures for classifying these RNA-seq samples in the same way as these samples had been classified using microarray data. Our results produced a 32-gene RNA-seq classifier which was 86.07% accurate and the most consistent with microarray-based classifications. This was an improvement of 16.4% on the model based on 793 shared genes.

The RNA-seq classifier and the GBM48 panel did not share any genes. This is likely because the genes that are the most informative and consistent across both microarray and RNA-seq platforms are not necessarily the most consistent and most informative genes when looking at microarray exclusively.

### Web Server for Classification

In order to facilitate the use of this algorithm, we built a web server at http://simplegbm.semel.ucla.edu/. This server supports microarray-based sample classification (one expression value per gene, submitted as a comma-separated file) and can produce outputs using the original Verhaak et al. classification scheme, as well as the GBM48 and RNA-seq panels. The server automatically handles normalization and batch effect adjustment utilizing ComBat [14]. In the case of RNA-seq data, rank normalization is utilized, which is better suited for cross-platform comparison with microarray data [37].

## Discussion

The goal of this project was to create a method as close in accuracy to the original classifier as possible, while using a significantly smaller number of variables. Centroid-based classifiers like ClaNC [3] and Prediction Analysis for Microarrays (PAM) [38] are excellent and intuitive ways of classifying groups; however, they rely upon gene-by-gene evaluations, i.e. each gene's fitness is evaluated on how well an individual gene separates out the different classifications. Gene-by-gene evaluation techniques tend to keep genes that have redundant information as they do not take into account multi-gene relationships or high levels of correlations between genes. By contrast, random forest models rely on decision trees, which allow multi-gene relationships to be accounted for in predictions. The multi-gene relationships permit fewer genes to better classify samples, and are a major benefit of utilizing random forest models in classification problems. Developing models on multi-gene relationships also has the benefit of potentially uncovering complex relationships between variables that would not be discoverable by evaluating each variable’s fitness independently.

Several popular methods for selecting a smaller subset of genes exist, including iterativeBMA [39], varSelRF [19], RFE [20] and R-SVM [40]. Of these, only varSelRF and RFE are designed to work with random forest models. The most common application of RFE is with support vector machines as in the R-SVM package [40]. VarSelRF has been reported to require fewer genes, and perform as well as other methods such as support vector machines (SVM), Diagonal Linear Discriminant Analysis (DLDA) and k-nearest neighbors models (KNN) [19,22]. Our method outperforms varSelRF and RFE for this application.

Earlier attempts to reduce classifiers of disease states using random forest models have largely relied on the varSelRF package in R [19]. One study of particular relevance which utilized varSelRF for variable selection with GBMs also attempted to re-evaluate the original classification technique using gene-isoform-based descriptors and random forest techniques [41]. However, the study produced an alternative classifier with low similarity to the original classifier, predicting the original classifications with only 81% accuracy. By comparison, our average random model using 121 randomly selected gene signatures was 85.60% accurate (1000 random models on our cohort of 803 samples). These random gene selections significantly outperformed the genes selected by this study. This is similar to our findings that the VarSelRF technique selects genes with similar accuracy to random models within our selected GBM datasets (84.51% on average for our 1000 test runs on our cohort of 803 samples). RFE fares only slightly better, presenting the need for an alternative strategy for gene subset selection.

A significant benefit of our hybrid method is that it allows the evaluation of subsets of probes at a time, allowing probes without complex relationships to be slowly and selectively removed. While many other algorithms include weighting by LDA and individual performance of genes, individual performance in our algorithm is completely ignored in favor of genes that work best in groups of a specified size. The advantage of specifying a set number of genes is that this allows for optimal groupings of genes to be selected in order to function within the confines of different diagnostic technologies that may require a limited number of genes in order to be practical.

Our GBM48 panel approximates the accuracy of the Verhaak et al. classifier while requiring expression values for 6% of the genes required by the original classifier. The Verhaak et al. classification itself was not perfect, as there was a distinction between 'core' samples and other samples that did not fit their classification as conferred by k-means clustering [1]. The core samples were selected through the use of silhouettes [42]. Negative silhouettes were given to 27 of the 200 samples, indicating that they did not fit their assigned classification. This can be interpreted as an accuracy rate of 86.5% for the original classification scheme and that approximately 13.5% of samples do not fit this classification scheme. Assuming the rest of the samples were randomly assigned classifications (3.38% of samples), it is possible to infer that the highest accuracy that can be achieved is 89.88% if the TCGA tumor training set used to build the Verhaak et al. model is representative of all GBMs. In our case, we considered our final result of 90.91% of samples being accurately assigned to be in the range of what would be expected from an excellent model. Additionally, the method design and use of data from multiple platforms, multiple batches and multiple laboratories ensures that only genes with the highest level of consistency are used in our final models.

In addition to the similarities in accuracy between our two models, we have also presented here in Fig 3 that our classifier has very similar survival curves to the original classification technique. This is presented as a benchmark for comparing clinical significance of our GBM48 panel with that of the Verhaak et al. classifier. We would like to also point out that the Verhaak et al. classification technique is not the best indicator of survival in GBM patients, despite favorable outcomes for patients diagnosed with the Proneural subtype. In fact, one of the best indicators of a favorable outcome for survival in patients undergoing treatment with temozolomide chemotherapy has been shown be the methylation of the MGMT promoter [43]. Additionally, at least one alternative strategy to classification has been proposed involving epigenetics. This integrative approach utilizes all available epigenetic, copy number variation, microarray expression and genetic variation instead of microarray data exclusively as a classification technique for GBMs, which shows the potential for improvement upon existing classification techniques [10].

The GARF framework presented here can be used for any project that requires the optimization of a model using a specific number of genes to work with a particular infrastructure, and likely would work with any other dataset with quantitative descriptors where variable reduction would be advantageous at the cost of a small amount of accuracy.

## Supporting Information

S1 Fig Statistically Significant Over-Enriched GO Terms from GOrilla. All statistically significant (FDR-corrected p-value <0.05) over-enriched GO terms are shown in this pathway in a hierarchical fashion as output from the GOrilla web server.
(TIF)

S1 Table VarselRF accuracy (1000 runs) on different datasets. Training represents 575 samples consisting of all TCGA samples and the three Gene Expression Omnibus (GEO) datasets used to direct the training process. Test represents the Rembrandt dataset (228 GBM samples).
(XLSX)

S2 Table Recursive feature selection (RFE) accuracy (1000 runs) on different datasets. Training represents 575 samples consisting of all TCGA samples and the three Gene Expression Omnibus (GEO) datasets used to direct the training process. Test represents the Rembrandt dataset (228 GBM samples).
(XLSX)
