# Supplementary figures and images for: Identification of an Efficient Gene Expression Panel for Glioblastoma Classification
Source: PLoS One. 2016 Nov 17;11(11):e0164649. doi: 10.1371/journal.pone.0164649 (PMC5113897; doi:10.1371/journal.pone.0164649)

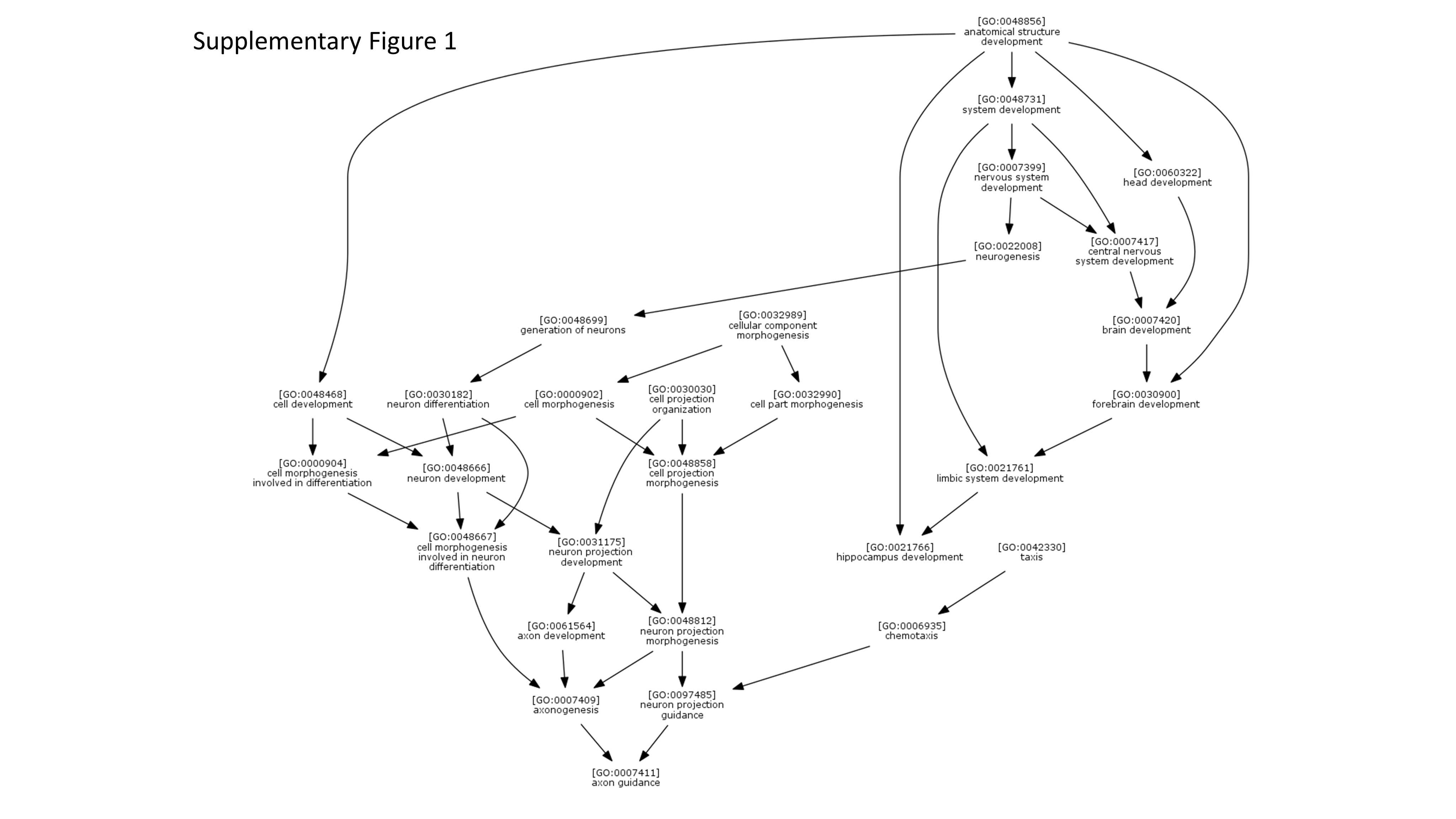

Supplement: S1 Fig — All statistically significant (FDR-corrected p-value <0.05) over-enriched GO terms are shown in this pathway in a hierarchical fashion as output from the GOrilla web server. (TIF) [file pone.0164649.s001.tif]
